# Postsurgical Otolaryngology Emergencies: A Simulation to Improve Multidisciplinary Patient Care During Rare, Critical Situations

**DOI:** 10.15766/mep_2374-8265.11612

**Published:** 2026-06-23

**Authors:** Andrew J. Neevel, Fatemeh Ramazani, Kaitlin Vance, Marie Leginza, Keith A. Casper, Marc C. Thorne, Robbi A. Kupfer

**Affiliations:** 1 Resident, Department of Otolaryngology-Head and Neck Surgery, University of Michigan Medical School; 2 Fellow, Department of Otolaryngology-Head and Neck Surgery, University of Michigan Medical School; 3 Nurse Practitioner, Department of Otolaryngology-Head and Neck Surgery, University of Michigan Medical School; 4 Clinical Associate Professor, Department of Otolaryngology-Head and Neck Surgery, University of Michigan Medical School; 5 Clinical Professor, Department of Otolaryngology-Head and Neck Surgery, University of Michigan Medical School

**Keywords:** Simulation, Otolaryngology, Airway, Tracheostomy

## Abstract

**Introduction:**

Tracheostomy false passage and carotid blowouts are rare otolaryngologic emergencies that require rapid identification and multidisciplinary management. This multidisciplinary simulation curriculum was designed to improve technical and nontechnical skills of otolaryngology nursing and medical teams.

**Methods:**

Multidisciplinary teams comprising nurses, medical students, and otolaryngology residents participated in 2 simulation scenarios featuring a high-fidelity mannequin, 3D-printed anterior neck, and standard airway equipment. Both cases involved a postoperative male patient (aged 50–60 years) following complex ablation and reconstruction for head and neck cancer. Scenarios simulated a carotid blowout and tracheostomy false passage (positioning outside of tracheal lumen). Participants completed pre- and postsimulation surveys, assessing self-perceived technical and nontechnical skills. Paired *t* tests were used to compare mean changes.

**Results:**

A total of 62 participants completed the simulation, the majority of whom were nurses (82%), along with medical students and otolaryngology residents, reflecting a multidisciplinary learner group. Mean nontechnical self-efficacy scores (assessing situational awareness, decision-making, communication/teamwork, and leadership) increased by 0.3 points on a 4-point scale postsimulation (*P* < .001). Technical self-efficacy increased by 0.5 points on a 5-point scale postsimulation (*P* < .001). Participants most cited improved teamwork (*n* = 23, 37%) and exposure to rare clinical scenarios (*n* = 19, 31%) as key benefits.

**Discussion:**

Carotid blowout and tracheostomy false passage simulations offer valuable exposure and practice in managing rare otolaryngology emergencies. Team-based interprofessional simulation improves technical and nontechnical self-efficacy and was positively received by participants.

## Educational Objectives

By the end of this activity, learners will be able to:

1. Recognize symptoms, consider relevant anatomy, and perform life-saving techniques necessary for management of otolaryngology postoperative emergencies (tracheostomy false passage, carotid blowout).
2. Articulate specific role in management of postoperative emergencies and understand adjacent team members’ roles and responsibilities.
3. Communicate effectively with nursing or otolaryngology providers to triage case acuity, recruit critical team members, and coordinate efficient team-based management of postoperative emergencies.

## Introduction

Postoperative otolaryngology patients present unique care challenges due to the risk of rare but potentially fatal complications requiring advanced airway management. In many centers, these patients receive care in specialized units with experienced nurses and on-call otolaryngology providers. However, critical complications such as carotid blowout and tracheostomy false passage occur so infrequently that even experienced providers may not encounter them in practice.^1,2^ Prompt recognition and coordinated interprofessional management are essential to reduce morbidity and mortality.^2–5^ These otolaryngology-specific complications require both technical and interprofessional communication skills that are rarely addressed in nursing, medical school, and postgraduate curricula.

There is a paucity of simulation curricula developed to teach and practice the technical and multidisciplinary elements of care required to manage these otolaryngology-specific emergencies. Existing simulation curricula focus on common clinical scenarios, such as airway obstruction, epistaxis, posttonsillectomy bleeding, neck trauma, pediatric difficult airway, and postthyroidectomy hematoma, as well as rare scenarios, such as tracheoinnominate fistula and cavernous carotid injury.^6–12^ Other broader curricula to develop otolaryngology knowledge and skills focus on a specific participant—otolaryngology resident, emergency medicine resident, medical student, or nursing—but not on their collaborative effort to manage.^13–16^ These simulations have been shown to improve both simulated and self-perceived comfort with management of these otolaryngologic emergencies.^8–16^ Further, simulation for rare emergencies has improved actual patient outcomes.^17,18^ While each unique emergency requires specific technical skills, the nontechnical domains of situational awareness, decision-making, communication, and leadership apply broadly across clinical practice. Multidisciplinary care is paramount for effective management in practice, particularly in critical emergencies.

Interprofessional simulation-based training in airway emergencies has been shown to enhance knowledge, technical skills, and nontechnical skills.^18,19^ We considered multiple educational frameworks described in simulation—behavioral, social cognitive, and constructivist—with the goal to provide an opportunity for deliberate practice, feedback from facilitators, and increased confidence and self-efficacy.^20,21^ To address this perceived gap in training for both technical and nontechnical skills in the otolaryngology inpatient setting, we developed a multidisciplinary simulation session focused on 2 rare but high-acuity scenarios: carotid blowout and tracheostomy false passage. Our goal was to create an opportunity for multidisciplinary collaboration to hone efficient response and management in these critical, time-sensitive, and life-threatening situations. Foundational skills, such as tracheostomy tube removal, suction, and initial management of alcohol withdrawal, were also embedded within the session.

## Methods

We developed a simulation-based curriculum targeting multidisciplinary teams involved in inpatient airway emergency management. The otolaryngology and nursing administrative leaders jointly selected the scenarios based on hospital risk management data, recent incidences, and multidisciplinary discussion. Carotid blowout and tracheostomy false passage were chosen, as they represent rare but high-consequence complications that demand rapid, coordinated management. Learning objectives were tailored specifically for nursing and medical participants through collaboration between otolaryngology faculty and experienced head and neck nurse practitioners (Appendices A and B). Participants were required to have baseline training appropriate to their professional practice; however, prior experience in airway or head and neck cancer–specific care was not required. Foundational skills, such as tracheostomy tube removal, suction, and initial management of alcohol withdrawal, were not separately taught or formally assessed; rather, they were expected baseline competencies for participating learners. The simulation scenarios provided an opportunity to observe how learners applied these skills in context, with feedback delivered during the debrief. Gaps in skills and knowledge were addressed during the facilitated debriefing, where participants analyzed their actions and identified opportunities for improvement with guidance from faculty facilitators.

### Equipment/Environment

Simulations were conducted in a dedicated clinical simulation center, designed to simulate an inpatient room. The setup included:

• Monitoring equipment
  ○ Cardiac monitor
  ○ Pulse oximeter
  ○ Temperature probe
  ○ Noninvasive blood pressure cuff
• Mannequin (Figure)
  ○ Option 1: Custom
    ▪ Torso mannequin with continuous airway and bag lungs
    ▪ 3D-printed laryngeal cartilage and trachea with anterior opening at site of tracheostomy
    ▪ Anterior neck opened to place 3D-printed trachea
    ▪ Velcro skin placed over anterior neck and 3D-printed trachea
  ○ Option 2: Gaumard HAL high-fidelity airway simulation mannequin
• Airway and procedural equipment
  ○ Stethoscope
  ○ Suction tubing
  ○ Noninvasive oxygenation/ventilation supplies: bag mask, non-rebreather mask, nasal cannula, oxygen tubing
  ○ Oral/nasal airways
  ○ Laryngoscope (Miller, Mac)
  ○ Flexible laryngoscope with light
  ○ Endotracheal intubation supplies: endotracheal tubes of various sizes, stylet, syringe
  ○ Scissors
  ○ Tracheostomy supplies: tube set (cuffed 6–0, obturator, inner cannula)
  ○ CO_2_ detector
  ○ Tape

**Figure.**
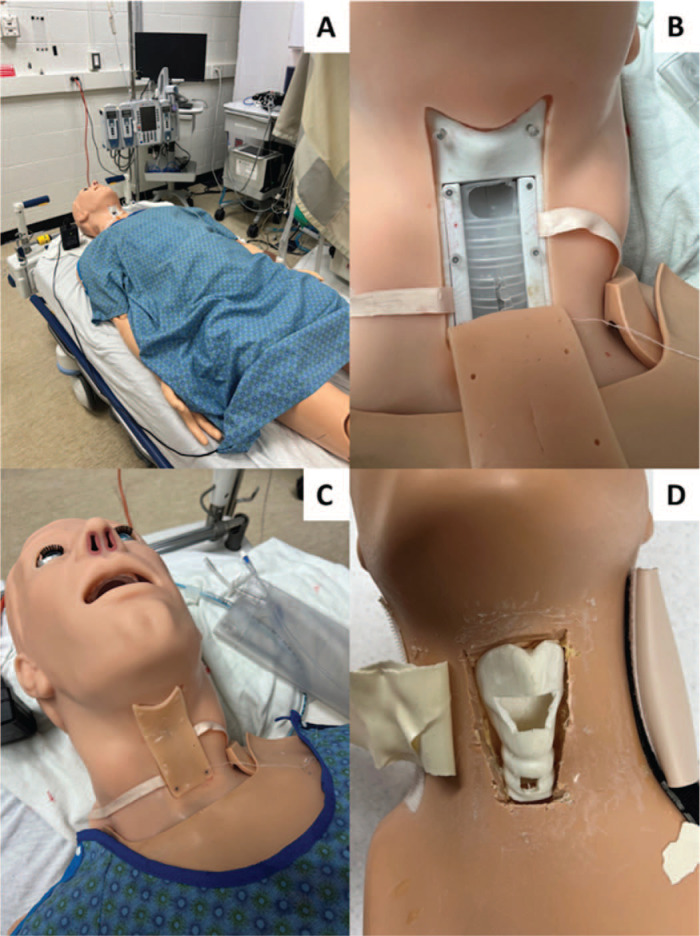
Mannequin setup. (A-C) High-fidelity airway mannequin (Gaumard HAL) with replaceable anterior neck skin. (D) Alternate custom airway with 3D-printed larynx for use in other mannequins.

### Personnel

Each simulation session was supported by 2 trained facilitators: 1 otolaryngology faculty member and 1 experienced nurse educator. All facilitators had prior experience in simulation-based education and completed a standardized orientation to the scenario content and debriefing structure before the simulation day to ensure consistency across sessions. Each session included 4 participant roles designed to reflect real-world interprofessional workflows: primary nurse, charge nurse, otolaryngology primary call provider (junior resident or nurse practitioner), and otolaryngology chief resident. These roles were intentionally selected to mirror typical chains of communication and clinical responsibility during postoperative emergencies. Learners rotated through junior and senior roles across sessions, allowing them to observe the scenario from different vantage points, gather information at varying stages of clinical escalation, and practice communicating effectively across levels of responsibility.

Each scenario was run by 2 facilitators: 1 otolaryngology faculty member and 1 experienced nurse educator. All facilitators, whether otolaryngology faculty or nurse educators, had prior experience and training in simulation facilitation and received a standardized orientation to the simulation scenarios and debriefing structure on the day of simulation. This ensured consistency in scenario delivery, learner support, and debrief quality across all sessions.

### Implementation

The simulation was implemented as a 2-hour session consisting of two 45-minute scenarios. Each scenario included a brief 5–10-minute prebrief for introductions and role clarification. The 2 scenarios ran concurrently in separate rooms, each staffed by 2 trained facilitators. Participants were divided into 2 groups. Each group completed 1 scenario followed by a 15-minute debrief in the same room, then switched rooms to complete the second scenario and its debrief. This structure allowed all learners to rotate through both active clinical roles and observer roles within a single 2-hour session. Across the 2 implementation dates, the full 2-scenario session was repeated 12 times to accommodate all participants. The simulation was implemented over 2 consecutive days to allow full participation of the inpatient otolaryngology nursing/medical staff during a scheduled mandatory unit educational retreat. Nurses were assigned to attend on 1 of the 2 days, ensuring adequate staffing in the clinical unit while allowing all nurses to participate during their paid workday. Otolaryngology residents and fourth-year medical students on service were invited to join the simulations; those who volunteered were released from clinical duties in brief rotating intervals to minimize disruption to patient care.

Each simulation team consisted of 5–7 learners: 4–5 nurses and 1–2 otolaryngology medical students and residents. Role assignments consist of a primary nurse, a secondary nurse, a primary otolaryngology provider, and a secondary otolaryngology provider, with all additional participants assigned to structured observer roles. Nurses and providers rotated through the primary, secondary, and observer roles across scenarios, allowing them to experience different communication demands and workflow perspectives. Otolaryngology residents and medical students participated in the provider roles appropriate to their training level, whereas remaining team members served as observers focusing on specific elements of team performance, such as communication, role clarity, and situational awareness.

Each scenario was conducted in a separate room, fully equipped with a 3D-printed anterior airway placed in a mannequin head, monitoring equipment, and all supplies required for diagnosis and management (Appendices C and D). Otolaryngology faculty and nurse educators facilitated the sessions together (see Appendices C and D) and conducted a 15-minute structured postsimulation debrief modeled after the Promoting Excellence and Reflective Learning in Simulation (PEARLS) Healthcare Debriefing Tool (Appendices E and F).^22^ Participants were encouraged to act as they would in real clinical practice. Facilitators supported this natural decision-making but guided teams back to the planned scenario flow when needed so that all key learning objectives were addressed. Pre- and postsimulation surveys were administered immediately before and after each session (Appendix G). The technical instrument was adapted from self-efficacy assessments validated for otolaryngology interns and medical students performing flexible laryngoscopy, peritonsillar abscess drainage, and myringotomy tube insertion and are routinely employed in simulation-based training at our institution.^23^ The nontechnical instrument utilized nontechnical skill categories validated in surgical education literature but was adapted for a broader range of learners (including nursing) and assessed self-efficacy instead of observed behavior.^24^ The total time required for the 2 scenarios and debriefs was 2 hours.

### Debriefing

Postsession debriefing proceeded with each individual group using a reflective model structured according to the PEARLS Healthcare Debriefing Tool, beginning with an introduction to the debrief and then participants’ individual reflections on the session, followed by a review of objectives. Facilitators guided the debrief discussion using a structured template, adapting the content based on participant insights of guiding the discussion as needed (see Appendices E and F). We recommend that an otolaryngology faculty member and an experienced nurse educator be available for the debrief discussion due to the complex and rare nature of these clinical scenarios. The presence of both practitioners will also allow for depth of multidisciplinary discussion.

### Assessment

Participants completed a self-assessment to assess whether the sessions’ stated educational objectives had been met. Pre- and postsimulation surveys assessed self-perceived technical and nontechnical competencies on a Likert scale (see Appendix G). Mean pre- and postsimulation competency scores were compared using paired *t* tests (IBM SPSS). Free-text responses were inductively coded to identify common themes related to the perceived value of the simulation. Feedback on the least valuable components of the session, as well as areas for improvement, were similarly collected using inductive coding. This initiative was deemed exempt from direct oversight after review by the University of Michigan Institutional Review Board (HUM00148692).

## Results

### Population

This curriculum was conducted over a single 2-hour session and included 62 participants, all of whom completed pre- and postsession surveys. Across the 2 training days, the curriculum was delivered in six 2-hour sessions, accommodating approximately 5–7 participants per session. In each session, 4 learners (2 nurses and 2 medical providers) participated in active “hot seat” roles, whereas the remaining 1–3 participants served as structured observers. A majority of participants were nurses (*n* = 51, 82%), with 6 medical students (10%) and 5 otolaryngology residents (8%).

### Nontechnical Skills

The mean overall nontechnical self-efficacy score (4-point scale)—comprising situational awareness, decision-making, communication/teamwork, and leadership—increased from 1.8 (95% CI :1.8-1.9) presimulation to 2.1 (95% CI: 2.0-2.1) postsimulation (*P* < .001). Each of the 4 individual domains demonstrated statistically significant improvement following the simulation session (Table 1).

**Table 1.**
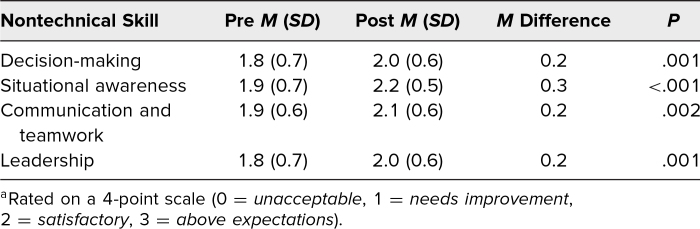
Mean Nontechnical Skill Confidence^a^ Pre- and Postsimulation (*N* = 61)

### Technical Skills

The mean overall technical self-efficacy score (based on a 5-point Likert scale)—including confidence in tracheostomy suctioning, tracheostomy removal, false passage management, alcohol withdrawal management, and carotid blowout management—increased from 3.8 (95% CI: 3.7-3.9) to 4.3 (95% CI: 4.2-4.3) following the session (*P* < .001). Confidence with each skill demonstrated statistically significant improvement when independently compared in subanalysis, except for tracheostomy suctioning (Table 2). Self-efficacy in managing tracheostomy false passage had the greatest improvement postsimulation.

**Table 2.**
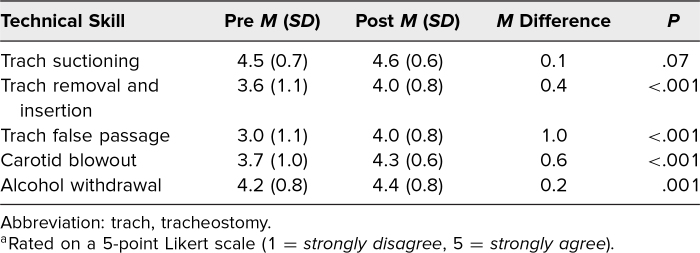
Mean Technical Skill Confidence^a^ Pre- and Postsimulation (*N* = 62)

### Participant Reflections

Qualitative analysis of free-text responses revealed that participants most valued the opportunity to practice teamwork (*n* = 23, 37%), engaging in realistic and clinically relevant scenarios (*n* = 19, 31%), receiving instruction and feedback from otolaryngologists (*n* = 12, 20%), and participating in a structured debrief session (*n* = 11, 18%). Suggested improvements included conducting simulation in a familiar clinical environment, more accurately simulating the wider context (the initial call, entrance, and introduction to the room and patient), and creating additional scenarios for more practice.

## Discussion

This multidisciplinary simulation curriculum, developed to improve management of rare inpatient otolaryngology emergencies in postoperative patients, led to significant improvements in participants’ self-perceived nontechnical and technical skills when managing carotid blowouts and tracheostomy false passage. Confidence improved across all technical domains, except tracheostomy suctioning, for which baseline confidence was already high. As expected, the greatest improvements were in management of tracheostomy false passage and carotid blowout, reflecting participants’ limited prior exposure to these clinical scenarios and the value of simulation exposure. Qualitative feedback highlighted participants’ appreciation of interprofessional collaboration, scenario realism, and debriefing, reinforcing the utility and relevance of this training.

These findings are consistent with previous simulation-based education studies in otolaryngology, demonstrating the value of simulation in training and education.^8–16^ Amin and Friedmann^8^ demonstrated a postsimulation improvement in perceived leadership abilities among participants who met the objectives of the airway emergency simulation course. Goldman et al.^9^ demonstrated a 1.9 point increase in confidence for pediatric airway management following simulation (based on a 1–5 Likert scale). This 1.9-point improvement is greater than the 0.5 achieved in this study, which can be credited to our score demonstrating an average across multiple simulations with a greater presimulation self-efficacy. La Monte et al.^13^ also described increased confidence in knowledge, teamwork, and manual skills (the largest increase) via a simulation workshop for junior residents. Similarly, Smith et al.^10^ compared lecture- and simulation-based programs and found significantly improved self-efficacy and oral examination performance after epistaxis and epiglottitis simulations. Together, these studies support the use of simulation as a useful strategy to improve preparedness for management of otolaryngologic emergencies.

Our study used a single-institution, small convenience sample, with unequal representation in residents compared to nurses, limiting generalization and conclusions of simulation efficacy for residents. It is also limited by its reliance on self-efficacy assessments, which are subject to confirmation bias and may not reflect objective skill acquisition. However, other otolaryngology simulation studies have demonstrated simultaneous improvement in self-efficacy and blind assessment.^15,25,26^ Structured observations and assessment, oral examinations, or validated assessment tools such as the nontechnical skills scale (NOTECHS) and nontechnical skills for surgeons (NOTSS) would offer a more rigorous assessment of participant competency.^8,10,24,27–29^ While we demonstrated an improvement in participant self-efficacy, nontechnical skills are challenging to teach and assess accurately, and simulation alone may not provide a sufficient framework without prior instruction.^30,31^ Further, our simplified nontechnical scale (scale 0–3, see Appendix G) may lack the resolution to quantify the benefit of this simulation. More comprehensive curricular initiatives should include foundational teaching in nontechnical domains, with simulation serving as a useful adjunct for education and assessment. Strategic timing of multiple self-efficacy assessments could also compare the educational benefit of various approaches (eg, didactics, case scenario discussion, and simulation). These scenarios are incorporated into larger-scale simulation “boot camps” for junior otolaryngology residents across the country; however, the simulations involve resident learners only and therefore lack the benefits of interprofessional team training.^13,16^ These complications are additionally covered in the Otolaryngology Core Curriculum, developed by the American Academy of Otolaryngology–Head and Neck Surgery, which is used as a framework for weekly didactics sessions in most otolaryngology residency programs. Future iterations that provide structured education sessions that could be provided to all nurses who care for patients with tracheostomies and head and neck cancer would strengthen the impact of this work.

Because these complications are rare, direct measurement of patient outcomes was not feasible within the scope of this project. However, simulation-based training is well established in the literature as an effective method for improving clinical preparedness, team communication, and patient safety. Broader implementation across additional centers would allow future studies to examine long-term clinical impact, but the current findings support the value of this training in strengthening readiness for high-risk, low-frequency events.

## Appendices


Scenario 1 Objectives.docxScenario 2 Objectives.docxScenario 1 Case.docxScenario 2 Case.docxScenario 1 Debrief.docxScenario 2 Debrief.docxPre- and Postsimulation Survey.docx
*All appendices are peer reviewed as integral parts of the Original Publication.*
