# Supplementary material for: Postsurgical Otolaryngology Emergencies: A Simulation to Improve Multidisciplinary Patient Care During Rare, Critical Situations
Source: MedEdPORTAL. 2026 Jun 23;22:11612. doi: 10.15766/mep_2374-8265.11612 (PMC13287035; doi:10.15766/mep_2374-8265.11612)
Supplement: Supplementary file 1 — Scenario 1 Objectives.docxScenario 2 Objectives.docxScenario 1 Case.docxScenario 2 Case.docxScenario 1 Debrief.docxScenario 2 Debrief.docxPre- and Postsimulation Survey.docx [file mep_2374-8265.11612-s001.zip › B. Scenario 2 Objectives.docx]

**Appendix B: Scenario Case and Objectives**

The case and objectives should be reviewed by all facilitators prior to performing the simulation. We recommend that the participants are not given this information prior to the simulation to maintain a diagnostic challenge and to promote active team-based problem-solving. This can be provided to participants during the debrief or the post-survey to facilitate self-reflection or to review after the session.

Case Title: Carotid Blowout

Case Summary: 64 y.o. man status post mandibulectomy for oral squamous cell carcinoma (SCCA); left neck dissection; radial forearm free flap reconstruction; tracheostomy; complicated by carotid blowout on POD#2.

Target Learners: Inpatient nurses, otolaryngology providers

Key points:

1. Evaluate bleeding in a post-surgical head and neck cancer patient.
2. Recognize a sentinel bleed and carotid blowout.
3. Appropriately manage carotid blowout situation: communication with team members, airway protection, equipment/monitoring, mobilize for operating room (OR)/interventional radiology (IR).

| **Learning Objectives: Nurses** |
| --- |
| **Knowledge:**   1. Develop a systematic approach to the evaluation of bleeding in a head and neck cancer patient. 2. Triage the severity of the bleeding, assess hemodynamic status and airway. 3. Recognize possible carotid blowout and understand management. 4. List equipment and management steps needed for management of carotid blowout (call staff assist, stat page service, rapid response team (RRT), get crash cart, tele pads, 2 large bore IVs, blood, airway protection, prepare to mobilize for OR/IR). |
| **Skills:**   1. Lavage and suction tracheostomy following hemoptysis. 2. Hold pressure at site of bleeding. 3. Inflate tracheostomy cuff. 4. Communicate effectively with otolaryngology team to explain the situation and acuity. |
| **Behavior:** Effective management of patient with hemoptysis and neck hemorrhage, appropriate triage of case acuity, and when and who to call for backup. |

| **Learning Objectives: Otolaryngology Providers** |
| --- |
| **Knowledge:**   1. Develop understanding of anterior neck anatomy related to trachs. 2. Develop a systematic approach to the evaluation of bleeding in a head and neck cancer patient. 3. Triage the severity of the bleeding, assess hemodynamic status and airway. 4. Recognize carotid blowout and understand management. 5. List equipment and management steps needed for management of carotid blowout (call senior and attending, get crash cart, 2 large bore IVs, blood, airway protection, prepare to mobilize for OR/IR). |
| **Skills:**   1. Inflate tracheostomy cuff if not already inflated upon arrival. 2. Hold pressure at sight of bleeding. 3. Communicate effectively with nursing team to determine the concern for carotid blowout. |
| **Behavior:** Effective management of patient with hemoptysis and neck hemorrhage, appropriate triage of case acuity, and when and who to call for backup. |
