# Supplementary material for: Postsurgical Otolaryngology Emergencies: A Simulation to Improve Multidisciplinary Patient Care During Rare, Critical Situations
Source: MedEdPORTAL. 2026 Jun 23;22:11612. doi: 10.15766/mep_2374-8265.11612 (PMC13287035; doi:10.15766/mep_2374-8265.11612)
Supplement: Supplementary file 1 — Scenario 1 Objectives.docxScenario 2 Objectives.docxScenario 1 Case.docxScenario 2 Case.docxScenario 1 Debrief.docxScenario 2 Debrief.docxPre- and Postsimulation Survey.docx [file mep_2374-8265.11612-s001.zip › C. Scenario 1 Case.docx]

Appendix C: Scenario 1 Case

Facilitators should review this document prior to starting the simulation. It includes details on initial presentation and simulation set-up. The facilitator operating the simulation mannikin or reporting vital signs and events may use the instructor notes as a recommended flow between events. When starting the simulation, the facilitator should read the history of present illness (HPI) to the participants and prompt them to ask questions about the history and physical exam.

| **Appendix A: *MedEdPORTAL* Simulation Case Template: Simulation 1**  SIMULATION CASE: Tracheostomy False Passage  AUTHORS: Andrew J. Neevel, MD, Kaitlin Vance, NP, Marie Leginza, NP, Keith A. Casper, MD, Marc C. Thorne, MD, MPH, FACS, Robbi A. Kupfer, MD  **LEARNER AUDIENCE:** Inpatient nurses, otolaryngology providers | |
| --- | --- |
| **PATIENT NAME:** Patient 1  **PATIENT AGE:** 58 years old  **CHIEF COMPLAINT:** Post-operative dyspnea and hypoxia  **PHYSICAL SETTING:** Inpatient floor | |
|  | |
| **Brief Narrative Description of Case** | A 58 y.o. man status post partial glossectomy for squamous cell carcinoma (SCCA) of the tongue with right radial forearm free flap reconstruction, tracheostomy (trach); complicated by tracheostomy false passage on post-operative day 2 in the setting of alcohol withdrawal.  Key points:   1. Evaluate dyspnea in a trach patient. 2. Work-up a trach with the inability to pass a suction catheter. 3. Recognize a tracheostomy false tract. 4. Develop techniques to optimize success of replacing a trach with a false tract. 5. Recognize alcohol withdrawal and initiate appropriate treatment. |
| **Primary Learning Objectives** | Otolaryngology providers:   1. Develop understanding of anterior neck anatomy related to tracheostomies. 2. Develop a systematic approach to the evaluation of dyspnea and obstruction in a tracheostomy patient. 3. Obtain a detailed history regarding why the trach is in place and how old it is. 4. Triage the acuity of the patient’s respiratory status. 5. Obtain necessary equipment for re-insertion and resuscitation (scissors, obturator, new trach, endotracheal tube (ETT), cricoid hook, flex scope, etc.). 6. Options to secure airway when unable to replace trach. 7. When to call your senior and attending for support. 8. Recognize and treat alcohol withdrawal.   Nurses:   1. Develop a systematic approach to the evaluation of dyspnea and obstruction in a tracheostomy patient. 2. Triage the acuity of the patient’s respiratory status. 3. Attempt to suction and apply appropriate monitoring and respiratory support (pulse oximetry, trach mask). 4. Recognize tracheostomy false passage 5. Obtain necessary equipment to assist in re-insertion and resuscitation (trach obturator, new trach set up, scissors, endotracheal tube, bag valve mask). 6. Recognize and treat alcohol withdrawal. |
| **Critical Actions** | Otolaryngology providers:   1. Communicate effectively with nursing team to determine the presence of tracheostomy false passage 2. Successfully replace tracheostomy   Nursing:   1. Suction tracheostomy. 2. Prepare new tracheostomy for insertion. 3. Communicate effectively with otolaryngology team to explain the situation and acuity. |
| **Learner Preparation or Prework** | No specific preparation or prework is required for this case. |

| Initial Presentation | | | |
| --- | --- | --- | --- |
| **Initial Vital Signs** | Heart Rate (HR): 110bpm  Blood Pressure (BP): 172/95 mmHg  Temperature: 37.1°C  Respiratory Rate (RR): 20 breaths/min  O_2_ saturation: 87-89%  Respiratory Pattern: tachypneic, stridor present | | |
| **Overall Setting and Appearance** | 1. Location: Inpatient Room 2. Monitors: Cardiac monitor, pulse oximetry, temperature probe, non-invasive blood pressure cuff 3. Mannequin position and attire: Wearing hospital gown, supine, agitated, with tremors, tracheostomy tube 4. Setting: On post-op day (POD) #2 patient appears anxious and restless at the beginning of the shift; earlier he threw his inner cannula at the nurse when they attempted to lavage and suction. He has been increasingly hypertensive and tachycardic. Patient puts on call light. When participants arrive, his inner cannula is on the floor; he is in a severe coughing fit and communicates that he cannot get enough air. | | |
| **Participants (and Their Roles in the Room at Case Start)** | All participants are non-standardized and assigned separate roles.   1. Primary nurse: You are the primary nurse for this patient on the inpatient floor. Patient puts on call light. When you arrive, his inner cannula is on the floor; he is in a severe coughing fit and communicates that he cannot get enough air. 2. Nursing staff/charge nurse: You respond to the staff assist. The patient’s primary nurse requests your help. 3. Otolaryngology primary call provider: You are the primary call provider for this patient, though are not familiar with his history. You receive a page from the nurse for a stat airway evaluation. 4. Otolaryngology senior resident: Your junior calls you with the acute airway details. They ask your assistance in management. | | |
| **HPI** | Given: A 58 y.o. man is post-operative day 2 after status post partial glossectomy for squamous cell carcinoma (SCCA) of the tongue with right radial forearm free flap reconstruction, tracheostomy (trach).  Offered when asked: alcohol abuse history, last drink one day prior to surgery | | |
| **Past Medical/Surgical History** | **Medications** | **Allergies** | **Family History** |
| PMH: Chronic obstructive pulmonary disease (COPD); Alcohol abuse; smoker; dysphagia; weight loss; depression; anxiety.  PSH: partial glossectomy for SCCA of the tongue with right radial forearm free flap reconstruction, tracheostomy, nasogastric placement, and right thigh skin graft used for arm donor site. | Atorvastatin  Albuterol | None | None |
| **Physical Examination** | | | |
| **General** | Alert, oriented, agitated | | |
| **HEENT** | Large well-perfused tissue present in oral cavity | | |
| **Neck** | Tracheostomy face plate sutured in place, small amount of blood around the stoma. | | |
| **Lungs** | Bilateral breath sounds present | | |
| **Cardiovascular** | Tachycardic, regular rhythm | | |
| **Abdomen** | Soft, compressible | | |
| **Neurological** | Cranial nerves and extremities grossly intact | | |
| **Skin** | Normal color and turgor. Radial forearm donor site dressed with wound vacuum and ace bandage | | |
| **GU** | No relevant findings | | |
| **Psychiatric** | Agitated | | |

Instructor Notes:

| Timing | Scenario Progression/Events | Expected Management | Management Pitfalls |
| --- | --- | --- | --- |
| Start | 1. Simulator Settings:  HR 100, BP 195/100, RR 20  O_2_ 88%. | Focused on initial evaluation of the patient’s airway and the acuity of the situation. Immediately put on staff assist. Attempt passing suction catheter. Place the patient on nasal oxygen supplementation. Recognize trach false passage and call service. | Failure to recognize the acuity of the situation given patient’s respiratory distress. Failure to put on staff assist. Failure to recognize trach false passage. Failure to call service. Failure to place the patient on oxygen supplementation. |
| 3 minutes | 2. Simulator Settings:  120, 195/100, 30, 80% | Recognize impending respiratory failure. Call senior resident. Communicate acuity of the situation, equipment and help needed. Understand options for airway management in current situation- replacement of trach versus orotracheal intubation. Consideration of replacement of trach via Seldinger technique or with ETT. Consideration of supplies to assist with stoma visualization. Obtain optimal patient positioning. | Failure to recognize the acuity. Failure to communicate effectively as a team. Failure to request help from senior resident. Failure to request necessary supplies. |
| 5 minutes | 3. Simulator Settings:  120, 195/100, 30, 75% | Cut sutures, remove tracheostomy and replace with or without Seldinger technique. Confirm accurate replacement with end tidal CO2, suction catheter, and/or scope. If unable to replace, consider using bougie or ETT. | Fail to proceed with trach removal and reinsertion. Failure to use bougie and/or ETT if unable to replace trach tube. |
| 10 minutes | 4. Simulator Settings:  120, 195/100, 15, 92% | Recognize continued hypertension and tachycardia as signs of alcohol withdrawal. Initiate institutional withdrawal protocol. Give IV lorazepam. | Failure to recognize alcohol withdrawal from agitation and vital signs. Failure to treat alcohol withdrawal appropriately. |

**Ideal Scenario Flow**

*Provide a detailed narrative description of the way this case should flow if participants perform in the ideal fashion.*

The nursing participant enters the room to find the patient in respiratory distress with their inner cannula on the ground. They recognized the patient is hypoxic and air is not flowing through the tracheostomy tube. They immediately put on staff assist and attempt passing suction catheter. They place the patient on nasal oxygen supplementation. They recognize possible trach false passage and call the service call provider. The provider arrives, received an efficient description by the bedside nurse, and assesses the patient. They call their senior resident, communicate acuity of the situation, as well as equipment and help needed. They quickly discuss options for airway management in current situation- replacement of trach versus orotracheal intubation. First, they obtain optimal patient positioning. They cut the sutures on the faceplate, remove the tracheostomy and replace with or without Seldinger technique. They confirm accurate replacement with end tidal CO2, suction catheter, and/or scope. If unable to replace, they consider using a bougie, scope or ETT, then confirm. Once the patient’s airway is secured, they recognize continued hypertension and tachycardia as signs of alcohol withdrawal. They discuss institutional withdrawal protocols with the nurse. They give IV lorazepam. The bedside nurse and otolaryngology primary call provider are the main participants but can enlist help from the charge or senior resident roles at any time.
