# Supplementary material for: Postsurgical Otolaryngology Emergencies: A Simulation to Improve Multidisciplinary Patient Care During Rare, Critical Situations
Source: MedEdPORTAL. 2026 Jun 23;22:11612. doi: 10.15766/mep_2374-8265.11612 (PMC13287035; doi:10.15766/mep_2374-8265.11612)
Supplement: Supplementary file 1 — Scenario 1 Objectives.docxScenario 2 Objectives.docxScenario 1 Case.docxScenario 2 Case.docxScenario 1 Debrief.docxScenario 2 Debrief.docxPre- and Postsimulation Survey.docx [file mep_2374-8265.11612-s001.zip › D. Scenario 2 Case.docx]

Appendix D: Scenario 2 Case

Facilitators should review this document prior to starting the simulation. It includes details on initial presentation and simulation set-up. The facilitator operating the simulation mannikin or reporting vital signs and events may use the instructor notes as a recommended flow between events. When starting the simulation, the facilitator should read the HPI to the participants and prompt them to ask questions about the history and physical exam.

| **Appendix A: *MedEdPORTAL* Simulation Case Template: Simulation 2**  SIMULATION CASE: Carotid Blowout  AUTHORS: Andrew J. Neevel, MD, Kaitlin Vance, NP, Marie Leginza, NP, Keith A. Casper, MD, Marc C. Thorne, MD, MPH, FACS, Robbi A. Kupfer, MD  **LEARNER AUDIENCE:** Inpatient nurses, otolaryngology providers | |
| --- | --- |
| **PATIENT NAME:** Patient 2  **PATIENT AGE:** 64 years old  **CHIEF COMPLAINT:** Post-operative tracheostomy bleeding  **PHYSICAL SETTING:** Inpatient floor | |
|  | |
| **Brief Narrative Description of Case** | A 64 y.o. man status post mandibulectomy for oral squamous cell carcinoma (SCCA); left neck dissection; radial forearm free flap reconstruction; tracheostomy; complicated by carotid blowout on POD#2.  Key points:   1. Evaluate bleeding in a post-surgical head and neck cancer patient. 2. Recognize a sentinel bleed and carotid blowout. 3. Appropriately manage carotid blowout situation: communication with team members, airway protection, equipment/monitoring, mobilize for operating room (OR)/interventional radiology (IR). |
| **Primary Learning Objectives** | Otolaryngology providers:   1. Develop understanding of anterior neck anatomy related to trachs. 2. Develop a systematic approach to the evaluation of bleeding in a head and neck cancer patient. 3. Triage the severity of the bleeding, assess hemodynamic status and airway. 4. Recognize carotid blowout and understand management. 5. List equipment and management steps needed for management of carotid blowout (call senior and attending, get crash cart, 2 large bore IVs, blood, airway protection, prepare to mobilize for OR/IR).   Nurses:   1. Develop a systematic approach to the evaluation of bleeding in a head and neck cancer patient. 2. Triage the severity of the bleeding, assess hemodynamic status and airway. 3. Recognize possible carotid blowout and understand management. 4. List equipment and management steps needed for management of carotid blowout (call staff assist, stat page service, rapid response team (RRT), get crash cart, tele pads, 2 large bore IVs, blood, airway protection, prepare to mobilize for OR/IR). |
| **Critical Actions** | Otolaryngology providers:   1. Inflate tracheostomy cuff if not already inflated upon arrival. 2. Hold pressure at sight of bleeding. 3. Communicate effectively with nursing team to determine the concern for carotid blowout.   Nursing:   1. Lavage and suction tracheostomy following hemoptysis. 2. Hold pressure at site of bleeding. 3. Inflate tracheostomy cuff.   Communicate effectively with otolaryngology team to explain the situation and acuity. |
| **Learner Preparation or Prework** | No specific preparation or prework is required for this case. |

| Initial Presentation | | | |
| --- | --- | --- | --- |
| **Initial Vital Signs** | Heart Rate (HR): 85bpm  Blood Pressure (BP): 136/78 mmHg  Temperature: 37.2°C  Respiratory Rate (RR): 18 breaths/min  O_2_ saturation: 94%  Respiratory Pattern: moving air, gurgling tracheal sounds, coughing | | |
| **Overall Setting and Appearance** | 1. Location: Inpatient Room 2. Monitors: Cardiac monitor, pulse oximetry, temperature probe, non-invasive blood pressure cuff 3. Mannequin position and attire: Wearing hospital gown, supine, agitated, with tremors, tracheostomy tube 4. Setting: On POD#2, the patient is using patient-controlled anesthesia frequently. The cuff of the trach was deflated this morning on rounds by the surgery service. You enter the room for shift assessment. Patient communicates need for tracheal suctioning. | | |
| **Participants (and Their Roles in the Room at Case Start)** | All participants are non-standardized and assigned separate roles.   1. Primary nurse: You are the primary nurse for this patient on the inpatient floor. You enter the room for shift assessment. Patient communicates need for tracheal suctioning. 2. Nursing staff/charge nurse: You respond to the staff assist. The patient’s primary nurse requests your help. 3. Otolaryngology primary call provider: You are the primary call provider for this patient, though are not familiar with his history. You receive a page from the nurse for a stat evaluation for bleeding. 4. Otolaryngology senior resident: Your junior calls you with the acute airway and bleeding details. They ask your assistance in management. | | |
| **HPI** | Given: A 64 y.o. man status post mandibulectomy for oral squamous cell carcinoma (SCCA); left neck dissection; radial forearm free flap reconstruction; tracheostomy.  Offered when asked: History of chemoradiation prior to salvage surgery | | |
| **Past Medical/Surgical History** | **Medications** | **Allergies** | **Family History** |
| PMH: chemoradiation to the head and neck, hypertension; high cholesterol; 2 pack-per-day smoker; alcohol abuse; depression; anxiety  PSH: mandibulectomy for oral squamous cell carcinoma (SCCA); left neck dissection; radial forearm free flap reconstruction; tracheostomy | Atorvastatin  Albuterol  Amlodipine | None | None |
| **Physical Examination** | | | |
| **General** | Alert, oriented, agitated | | |
| **HEENT** | Large well-perfused tissue present in oral cavity | | |
| **Neck** | Tracheostomy face plate sutured in place, significant amount of bright red blood around the stoma and expectorating through trach tube. | | |
| **Lungs** | Bilateral breath sounds present | | |
| **Cardiovascular** | Tachycardic, regular rhythm | | |
| **Abdomen** | Soft, compressible | | |
| **Neurological** | Cranial nerves and extremities grossly intact | | |
| **Skin** | Normal color and turgor. | | |
| **GU** | No relevant findings | | |
| **Psychiatric** | Agitated | | |

Instructor Notes:

| Timing | Scenario Progression/Events | Expected Management | Management Pitfalls |
| --- | --- | --- | --- |
| Start | 1. Simulator Settings:  HR 100, BP 148/75, RR 20  O_2_ 97%.  1. Hemoptysis from trach upon lavage.  2. Bleeding from neck incision.  3. Family member (played by faculty) is becoming very concerned about amount of bleeding. | Attempt to suction, notice neck incisional bleeding. Recognize that patient is on carotid blowout precautions, integrate that knowledge with assessment of the situation. Hold pressure at the site of bleeding and put on staff assist. Call service and code team. Inflate tracheostomy cuff to protect the airway. Obtain monitoring and resuscitation equipment (crash cart, IV fluids, blood, tele/EKG pads). Keep patient as calm as possible. | Failure to recognize the acuity of the situation given patient’s carotid blowout precautions order, hemoptysis, and persistent neck bleeding. Failure to call staff assist, service, and RRT once a blowout is recognized. Failure to stop bleeding by holding pressure. Failure to inflate trach cuff to protect airway. Failure to begin resuscitation. |
| 5 minutes | 2. Simulator Settings:  120, 140/68, 25, 95%  1. Service team arrives.  2. Bleeding of the neck is controlled with pressure by nurse.  3. Hemoptysis slows down after inflating trach cuff. | Recognize carotid blowout. Call senior resident, attending, and mobilize for OR/IR. Communicate acuity of the situation, equipment and help needed. Ensure airway is adequately protected. | Failure to recognize the acuity. Failure to communicate effectively as a team. Failure to request help from senior resident and attending. |
| 8 minutes | 3. Simulator Settings:  120, 122/54, 25, 95% | Calmly and clearly explain the situation to patient and family and the plan. Ensure patient is ready for direct transport to OR. Simulation is over when team is prepared for OR. | Failure to call OR for stat direct transport. Failure to prepare patient for transport (should ask for tele pads, monitor, chart). Failure to communicate to patient and family about the situation. |

**Ideal Scenario Flow**

*Provide a detailed narrative description of the way this case should flow if participants perform in the ideal fashion.*

The nursing participant enters the room to find the patient had significant hemoptysis from his trach. On further examination, he is oozing from his neck sutures. They attempt to suction through the trach, which yields additional bleeding. They hold pressure at the site of bleeding, put on staff assist, call service, and call the code or rapid resuscitation team. They inflate the tracheostomy cuff to protect the airway. They call for monitoring and resuscitation equipment (crash cart, IV fluids, blood, tele/EKG pads). They keep patient as calm as possible. Once the provider on call arrives, they recognize the possibility of carotid blowout and confirm the nurse’s first steps. They then call their senior resident, fellow/attending, and mobilize the patient for the operating room or call interventional radiology. They communicate the acuity of the situation, equipment and help needed. They continue to ensure airway is adequately protected. The provider or senior calmly and clearly explain the situation to patient and family and the plan. They ensure patient is ready for direct transport to OR.
