# Supplementary material for: Postsurgical Otolaryngology Emergencies: A Simulation to Improve Multidisciplinary Patient Care During Rare, Critical Situations
Source: MedEdPORTAL. 2026 Jun 23;22:11612. doi: 10.15766/mep_2374-8265.11612 (PMC13287035; doi:10.15766/mep_2374-8265.11612)
Supplement: Supplementary file 1 — Scenario 1 Objectives.docxScenario 2 Objectives.docxScenario 1 Case.docxScenario 2 Case.docxScenario 1 Debrief.docxScenario 2 Debrief.docxPre- and Postsimulation Survey.docx [file mep_2374-8265.11612-s001.zip › E. Scenario 1 Debrief.docx]

**Appendix E: Scenario 1 Debriefing Suggestions**

This debrief should be reviewed by all facilitators prior to the simulation. After the simulation is performed, use the Debrief Structure to lead the participant discussion. Knowledge and key points may be integrated into the discussion or reviewed at the end to ensure all important details are covered.

Debrief Structure, modified from the PEARLS Healthcare Debriefing Tool^1^

| **Step** | **Objective** | **Sample Phrases** |
| --- | --- | --- |
| 1. Setting the Scene | Create a safe context for learning | “Let’s spend 15 minutes debriefing. Our goal is to improve how we work together and take care of our patients.” |
| 1. Reactions | Solicit initial reactions and explore feelings | “Any initial reactions?” |
| 1. Description | Clarify facts | “What was the working diagnosis? Does everyone agree?” |
| 1. Analysis | Explore performance domains | **Preview Statement:** *(use to introduce new topic)*  “At this point, I'd like to spend some time talking about [insert topic, e.g. assessing respiratory distress in patients with a tracheostomy].”  **Mini Summary:** *(use to summarize discussion of one topic)*  “That was a great discussion. Are there any additional comments related to [topic or performance gap]?  “Any outstanding issues or concerns?” |
| 1. Application and Summary | Identify take-aways | Learner-centered: “What are some take-aways from this discussion for our clinical practice?”  Instructor-centered: “The key learning points for the case were [insert key points from list below].” |

^1^ Bajaj K, Meguerdichian M, Thoma B, Huang S, Eppich W, Cheng A. The PEARLS Healthcare Debriefing Tool. Acad Med. 2018 Feb;93(2):336. PMID: 29381495.

Knowledge:

1. Anterior neck anatomy and the risk of false passage anterior to the trachea
2. Special considerations for airway management when unable to pass a suction through a tracheostomy
3. Tools available to assist in replacement of an early post-operative tracheostomy
4. Early activation of additional help in an acute airway situation

Key Points:

1. With any airway issue in which the patient is in respiratory distress, staff assist should be called immediately and the service should be paged. Senior residents should be called immediately if patient is in respiratory distress. The age of the tracheostomy, reason for tracheostomy, and status of the patient’s upper airway (able to orotracheally intubate or not) should be communicated to the response team.
2. There is a variable amount of soft tissue anterior to the trachea through which a tracheostomy tube can be false passaged. In fresh post-operative tracheostomies, we employ Bjork flap to prevent this.
3. Inability to pass a suction catheter through a tracheostomy should concern you for possible false passage. This may also occur with an occlusive mucus plug or blood clot. Sometimes a tracheostomy tube is not seated well within the airway and the suction catheter may be hitting the anterior or posterior wall of the trachea.
4. Tools to assist with stoma exposure include: surgical retractors, a hemostat, a cric hook. Positioning is also key. Place a rolled towel or blanket under the patient’s upper back/neck to extend the neck, exposing the stoma. Make sure to have adequate lighting as well. If overhead lights are poor, assign someone in the room to hold a light for you or use a head light.
5. If tracheostomy cannot be replaced easily, use of Seldinger technique over a flexible scope or bougie is often helpful. An ETT can also be passed into the stoma and is typically easier to find the airway because it is more flexible and allows better visualization during placement.
6. If replacement of the tracheostomy tube has already been attempted, then there may be local tissue trauma and bleeding. Make sure you have suction available to clear any blood, clot or mucus for better visualization of the airway.
7. Once the trach is replaced, it is always good practice to confirm appropriate location with flexible tracheoscopy. Make sure to re-secure the trach tube and avoid additional trach changes for a few days to allow the false passaged tract to heal.
8. Agitation, tachycardia, and hypertension in a postoperative patient should raise concern for alcohol withdrawal, which can be life-threatening if untreated. Withdrawal symptoms may begin within 4-8 hours of the patient’s last drink and escalate until approximately 72 hours after last alcohol intake. Immediate treatment is IV lorazepam. Close monitoring and/or an institutional withdrawal protocol should be initiated for all patients in whom alcohol withdrawal is a concern.
