# Supplementary material for: Postsurgical Otolaryngology Emergencies: A Simulation to Improve Multidisciplinary Patient Care During Rare, Critical Situations
Source: MedEdPORTAL. 2026 Jun 23;22:11612. doi: 10.15766/mep_2374-8265.11612 (PMC13287035; doi:10.15766/mep_2374-8265.11612)
Supplement: Supplementary file 1 — Scenario 1 Objectives.docxScenario 2 Objectives.docxScenario 1 Case.docxScenario 2 Case.docxScenario 1 Debrief.docxScenario 2 Debrief.docxPre- and Postsimulation Survey.docx [file mep_2374-8265.11612-s001.zip › F. Scenario 2 Debrief.docx]

**Appendix F: Scenario 2 Debriefing Suggestions**

This debrief should be reviewed by all facilitators prior to the simulation. After the simulation is performed, use the Debrief Structure to lead the participant discussion. Knowledge and key points may be integrated into the discussion or reviewed at the end to ensure all important details are covered.

Debrief Structure, modified from the PEARLS Healthcare Debriefing Tool^1^

| **Step** | **Objective** | **Sample Phrases** |
| --- | --- | --- |
| 1. Setting the Scene | Create a safe context for learning | “Let’s spend 15 minutes debriefing. Our goal is to improve how we work together and take care of our patients.” |
| 1. Reactions | Solicit initial reactions and explore feelings | “Any initial reactions?” |
| 1. Description | Clarify facts | “What was the working diagnosis? Does everyone agree?” |
| 1. Analysis | Explore performance domains | **Preview Statement:** *(use to introduce new topic)*  “At this point, I'd like to spend some time talking about [insert topic, e.g. how to secure the airway in a patient with large volume bleeding in the head and neck].”  **Mini Summary:** *(use to summarize discussion of one topic)*  “That was a great discussion. Are there any additional comments related to [topic or performance gap]?  “Any outstanding issues or concerns?” |
| 1. Application and Summary | Identify take-aways | Learner-centered: “What are some take-aways from this discussion for our clinical practice?”  Instructor-centered: “The key learning points for the case were [insert key points from list below].” |

^1^ Bajaj K, Meguerdichian M, Thoma B, Huang S, Eppich W, Cheng A. The PEARLS Healthcare Debriefing Tool. Acad Med. 2018 Feb;93(2):336. PMID: 29381495.

Knowledge:

1. Factors that increase risk for carotid blowout.
2. Equipment and monitoring that should be available for patients on carotid blowout precautions.
3. Need to secure the airway with a cuffed trach tube when there is an uncontrolled source of large volume bleeding.
4. Early activation of additional help in a carotid blowout situation.
5. Understand the sources of bleeding and how they can be treated.

Key Points:

1. After the cuff is let down for the first time, there may be a small amount of hemoptysis. Large volume bright red blood is concerning for an active bleed. If the bleeding is uncontrolled, the cuff of the trach tube should be immediately inflated or tube should be exchanged for a cuffed tube to prevent aspiration of large volumes of blood, which can clot and cause proximal or distal airway obstruction.
2. Patients who have a history of chemoradiation and have other surgical factors that expose large blood vessels in the oropharynx and neck are at risk of a carotid blowout. The otolaryngology service will place these patients on carotid blowout precautions, which ensures that resuscitation equipment is at the bedside and that staff are aware of the risk.
3. Carotid blowouts may present with a “sentinel bleed” or a less dramatic bleeding event, which can progress to large, immediately life-threatening bleeding within minutes to hours. Suspicion for a sentinel bleed or carotid blowout must be very high for patients with risk factors.
4. With large volume bleeding, it may be difficult to determine the source. Knowledge of the patient’s tumor and/or surgical history can help identify potential sources. Bleeding from the trach may be coming from an oropharyngeal source, which could require intraoral packing to control. This is generally only possible if patient has a tracheostomy.
5. Patients with carotid blowout should be kept as calm as possible to prevent higher blood pressure and increased bleeding. It is important to stay calm and communicate clearly to the team and patient/family.
6. Definitive management, if possible, includes going to the OR and/or IR to control the bleeding. If the bleed is coming from the common or internal carotid, there is a high risk of stroke as a result of methods used to control the bleeding.
