# Supplementary material for: Postsurgical Otolaryngology Emergencies: A Simulation to Improve Multidisciplinary Patient Care During Rare, Critical Situations
Source: MedEdPORTAL. 2026 Jun 23;22:11612. doi: 10.15766/mep_2374-8265.11612 (PMC13287035; doi:10.15766/mep_2374-8265.11612)
Supplement: Supplementary file 1 — Scenario 1 Objectives.docxScenario 2 Objectives.docxScenario 1 Case.docxScenario 2 Case.docxScenario 1 Debrief.docxScenario 2 Debrief.docxPre- and Postsimulation Survey.docx [file mep_2374-8265.11612-s001.zip › G. Pre- and Postsimulation Survey.docx]

**Appendix G: Pre- and Post- Simulation Surveys**

This document may be provided to the participants to complete before the simulation begins and after the debrief. If facilitators prefer to adapt the simulations for a third-party observed technical skill evaluation, we recommend excluding the pre-simulation technical skill survey, as this indicates the case topics.

Self-reported Non-technical Skills Survey Administered Pre- and Post- Simulation

| **ASPECT OF CARE** | N/A  (-) | Unacceptable  (0) | Needs Improvement  (1) | Satisfactory  (2) | Above Expectations  (3) |
| --- | --- | --- | --- | --- | --- |
| **Situational Awareness**  My current ability to:   1. Gather information 2. Understand information 3. Project and anticipate future situations |  |  |  |  |  |
| **Decision Making**  My current ability to:   1. Consider options 2. Select and communicate options 3. Implement and review decisions |  |  |  |  |  |
| **Communication and Teamwork**  My current ability to:   1. Exchange information 2. Establish a shared understanding 3. Coordinate team activities |  |  |  |  |  |
| **Leadership**  My current ability to:   1. Set and maintain standards 2. Support others 3. Cope with pressure |  |  |  |  |  |

Self-reported Technical Skills Survey Administered Pre- and Post- Simulation

| **Prior to/After completion of this simulation, I am able to effectively perform/manage:** | **Strongly Disagree** | **Disagree** | **Neutral** | **Agree** | **Strongly Agree** |
| --- | --- | --- | --- | --- | --- |
| Tracheostomy suctioning | 1 | 2 | 3 | 4 | 5 |
| Tracheostomy removal and insertion | 1 | 2 | 3 | 4 | 5 |
| Tracheostomy false tract | 1 | 2 | 3 | 4 | 5 |
| Alcohol withdrawal in postop patient | 1 | 2 | 3 | 4 | 5 |
| Carotid blowout | 1 | 2 | 3 | 4 | 5 |
