# Supplementary material for: Postsurgical Otolaryngology Emergencies: A Simulation to Improve Multidisciplinary Patient Care During Rare, Critical Situations
Source: MedEdPORTAL. 2026 Jun 23;22:11612. doi: 10.15766/mep_2374-8265.11612 (PMC13287035; doi:10.15766/mep_2374-8265.11612)
Supplement: Supplementary file 1 — Scenario 1 Objectives.docxScenario 2 Objectives.docxScenario 1 Case.docxScenario 2 Case.docxScenario 1 Debrief.docxScenario 2 Debrief.docxPre- and Postsimulation Survey.docx [file mep_2374-8265.11612-s001.zip › A. Scenario 1 Objectives.docx]

**Appendix A: Scenario 1 Case and Objectives**

The case and objectives should be reviewed by all facilitators prior to performing the simulation. We recommend that the participants are not given this information prior to the simulation to maintain a diagnostic challenge and to promote active team-based problem-solving. This can be provided to participants during the debrief or the post-survey to facilitate self-reflection or to review after the session.

Case Title: Tracheostomy False Passage

Case Summary: A 58 y.o. man status post partial glossectomy for squamous cell carcinoma (SCCA) of the tongue with right radial forearm free flap reconstruction, tracheostomy (trach); complicated by tracheostomy false passage on post-operative day 2 in the setting of alcohol withdrawal.

Target Learners: Inpatient nurses, otolaryngology providers

Key points:

1. Evaluate dyspnea in a trach patient.
2. Work-up a trach with the inability to pass a suction catheter.
3. Recognize a tracheostomy false tract.
4. Develop techniques to optimize success of replacing a trach with a false tract.
5. Recognize alcohol withdrawal and initiate appropriate treatment.

| **Learning Objectives: Nurses** |
| --- |
| **Knowledge:**   1. Develop a systematic approach to the evaluation of dyspnea and obstruction in a tracheostomy patient. 2. Triage the acuity of the patient’s respiratory status. 3. Attempt to suction and apply appropriate monitoring and respiratory support (pulse oximetry, trach mask). 4. Recognize tracheostomy false passage 5. Obtain necessary equipment to assist in re-insertion and resuscitation (trach obturator, new trach set up, scissors, endotracheal tube, bag valve mask). 6. Recognize and treat alcohol withdrawal. 7. Understand Michigan Alcohol Withdrawal Severity (MAWS)* protocol and initiate it appropriately. |
| **Skills:**   1. Suction tracheostomy. 2. Prepare new tracheostomy for insertion. 3. Communicate effectively with otolaryngology team to explain the situation and acuity. |
| **Behavior:** Effective management of a patient in respiratory distress, appropriate triage of case acuity and when and who to call for backup. |

| **Learning Objectives: Otolaryngology Providers** |
| --- |
| **Knowledge:**   1. Develop understanding of anterior neck anatomy related to tracheostomies. 2. Develop a systematic approach to the evaluation of dyspnea and obstruction in a tracheostomy patient. 3. Obtain a detailed history regarding why the trach is in place and how old it is. 4. Triage the acuity of the patient’s respiratory status. 5. Obtain necessary equipment for re-insertion and resuscitation (scissors, obturator, new trach, endotracheal tube (ETT), cricoid hook, flex scope, etc.). 6. Options to secure airway when unable to replace trach. 7. When to call your senior and attending for support. 8. Recognize and treat alcohol withdrawal. 9. Understand MAWS* protocol and initiate it appropriately. |
| **Skills:**   1. Communicate effectively with nursing team to determine the presence of tracheostomy false passage 2. Successfully replace tracheostomy. |
| **Behavior:** Effective management of a patient in respiratory distress, appropriate triage of case acuity and when to call for backup. |

*The Michigan Alcohol Withdrawal Severity (MAWS) is a publicly available severity scale for assessing and safely managing non-critically ill patients with acute alcohol withdrawal in a non-intensive care setting. This is available in the National Library of Medicine (<https://www.ncbi.nlm.nih.gov/books/NBK604324/pdf/Bookshelf_NBK604324.pdf>). Other institutional withdrawal protocols could be substituted if preferred.
